# Angiogenesis in Pancreatic Cancer: Pre-Clinical and Clinical Studies

**DOI:** 10.3390/cancers11030381

**Published:** 2019-03-18

**Authors:** Tiziana Annese, Roberto Tamma, Simona Ruggieri, Domenico Ribatti

**Affiliations:** Department of Basic Medical Sciences, Neurosciences and Sensory Organs, Section of Human Anatomy and Histology, University of Bari Medical School Bari, 70124 Bari, Italy; tiziana.annese@uniba.it (T.A.); roberto.tamma@uniba.it (R.T.); simona.ruggieri@uniba.it (S.R.)

**Keywords:** angiogenesis, anti-angiogenic therapy, pancreatic cancer, tumor progression

## Abstract

Angiogenesis is a crucial event in tumor development and progression, occurring by different mechanisms and it is driven by pro- and anti-angiogenic molecules. Pancreatic cancer vascularization is characterized by a high microvascular density, impaired microvessel integrity and poor perfused vessels with heterogeneous distribution. In this review article, after a brief introduction on pancreatic cancer classification and on angiogenesis mechanisms involved in its progression, the pre-clinical and clinical trials conducted in pancreatic cancer treatment using anti-angiogenic inhibitors will be described. Finally, we will discuss the anti-angiogenic therapy paradox between the advantage to abolish vessel supply to block tumor growth and the disadvantage due to reduction of drug delivery at the same time. The purpose is to identify new anti-angiogenic molecules that may enhance treatment regimen.

## 1. Introduction

Pancreatic cancer is the seventh leading cause of cancer death in the world in 2018, with an incidence of 2.5% and a mortality of 4.5% [1]. It has a higher incidence in high/very high developed countries compare to low/medium developed countries [1]. Pancreatic cancer is asymptomatic until the disease reaches an advanced stage and this is one of the causes of the low survival rate. Indeed, there is no a screening program to improve prognosis through early diagnosis. Individuals with a family history of pancreatic cancer, as well as cigarette smokers, and suffering from chronic pancreatitis and diabetes mellitus have an increased risk of developing pancreatic cancer (see Table 1 for an overview of risk factors) [2,3,4,5].

World Human Organization classified pancreatic cancer in epithelial tumors, exocrine and neuroendocrine ones, mature teratoma, mesenchymal tumors, lymphomas and secondary tumors based on histologic features [6]. Usually, two big types are identified, such as cancers of exocrine gland and the cancers of endocrine one.

The major part (>95%) are cancers of exocrine gland and these are mostly (>85%) pancreatic ductal adenocarcinomas, that via lymph vascular system metastasize to organs, such as liver [7,8]. Curative treatment of pancreatic ductal adenocarcinomas is surgery, but only 10–15% of the patients present a resectable cancer mass and are candidate to subsequent adjuvant therapy to avoid local and systemic recurrence [9]. Indeed, pancreatic ductal adenocarcinoma is resistant to chemotherapy. Gemcitabine- or 5-Fluorouracil-based chemo-radiation were employed in either early stage or advanced pancreatic cancer with moderate survival benefits [10,11]. The most recent therapeutic protocols provide for the use of combined chemotherapeutic agents in association with agents targeting specific molecular pathways (i.e., fibroblast growth factor (FGF), vascular endothelial growth factor (VEGF), farnesyl-transferase inhibition) or the cellular or acellular components of fibrotic tumor stroma [12].

Cancers of the endocrine gland, also called pancreatic neuroendocrine tumors are less common (1–2% of all pancreatic cancer in Europe) and are most often benign [13]. Based on secretion of endocrine hormones, including gastrin, insulin, glucagon, or vasoactive-intestinal peptide, some types of pancreatic neuroendocrine tumors are functional and other are non-functional showing a wide heterogeneity with highly variable prognosis. For pancreatic neuroendocrine tumors the first line of attack is the surgical resection if the tumor is identified early and in a localized stage [14]. Pancreatic neuroendocrine tumors metastasize, especially in the liver [15,16,17]. In addition to surgery, control hormone-dependent symptom is mandatory [18]. Therefore, given the high heterogeneity of pancreatic cancer, an accurate characterization of different types by origin, degree and metastasis presence/absence and their localization, are crucial in order to adopt the most appropriate therapy.

## 2. Angiogenesis in Pancreatic Cancer

Angiogenesis is involved in tumor development and progression and plays a key role in development of metastasis [19]. It occurs by different mechanisms and it is driven by many pro- and anti-angiogenic molecules [20].

Li et al. exhaustively explained the different pathways of angiogenic and non-angiogenic types of vascularization in pancreatic cancer pointing out the possible mechanisms for the poor efficacy of anti-angiogenic therapies [21]. Among these, vessel co-option, vasculogenic mimicry and vasculogenesis are noteworthy, because they seem to play a key role in the ineffectiveness of classical anti-angiogenic therapies in pancreatic tumors, as they represent alternative and compensatory mechanisms of tumor growth and progression. Franco et al., by the use of a genetically engineered mouse model of pancreatic neuroendocrine tumors, demonstrated the utility of α-smooth muscle actin (α-SMA), expressed by pericytes surrounding tumor co-opted vessels, as surrogate marker for response or evasive resistance to anti-angiogenic therapy [22]. Yang et al., by hypoxia inducible factor 2 alpha (HIF-2α) immunohistochemistry on pancreatic cancer patients and by HIF-2α-induced vasculogenic mimicry in vitro and in vivo experiments, demonstrated that HIF-2α overexpression and vasculogenic mimicry are correlated with poor tumor differentiation, late clinical stage, lymph node metastasis, and poor prognosis [23]. Others authors demonstrated that endothelial progenitor cells contribute to pancreatic cancer vasculogenesis homing to tumor area under the stimulation of different pro-angiogenic factors released by pancreatic cancer cells [24,25,26,27,28].

Furthermore, pancreatic cancer is characterized by a high microvascular density, impaired microvessel integrity and poor perfused vessels with heterogeneous distribution in different subtypes of pancreatic cancer, even within a specific type [29,30]. High microvascular density together with low microvessel integrity are associated with early recurrence, metastasis and short survival after tumor resection [31]. These altered characteristics of the pancreatic cancer vasculature may be targets of therapies with the purpose to induce normalization of tumor blood vessels, resulting in improved tumor perfusion, reduced hypoxia, and improved drug delivery and therapeutic outcomes [32,33,34]. Yapp et al., comparing the efficacy of metronomic chemotherapy versus conventional chemotherapy regimen, showed that metronomic treatment affects both tumor vasculature and tumor pancreatic cells [35]. Indeed, vessel density increased, tumor perfusion was transiently improved, and hypoxia decreased [35].

Pancreatic cancer angiogenesis is activated by genetic and epigenetic alterations and by cells and stromal components of tumor microenvironment. Those diverse tumor inducers determine a limited sets of nuclear transcription factors, including Sp1, Stat3 and NF-κB [36], conferring a high survival and growth advantage to cancer cells through alteration of the expression and functions of downstream effector factors, such as VEGF and interleukin 8 (IL-8). Therefore, transcription factors should be considered for the development of new antitumor and anti-angiogenic therapeutic approaches.

Among the cells of the tumor microenvironment, pancreatic stellate cells, the major tumor stroma pro-fibrogenetic cells, together with pancreatic cancer cells, support the pro-angiogenic hypoxia microenvironment increasing the expression of endostatin, that in turn induce the expression of several pro-angiogenic molecules [37,38,39].

Moreover, mast cells, tumor associated macrophages and neutrophils were found to play a critical role in the regulation of pancreatic cancer microenvironment. In pancreatic ductal adenocarcinoma infiltrated mast cell counts and serum mast cells tryptase levels are positively correlated with tumor microvascular density indicating a worse prognosis [40,41]. Tumor associated macrophages, prevalent M2 likes, facilitate pancreatic cancer cells progression and migration in a VEGF-dependent manner [42]. M2 also have a central role in stimulating angiogenesis mediated by the expression on their membrane of folate receptor β-expressing, by their recruitment via HIF-1α–CCL2 pathway and by the pancreatic stellate cells activation [43,44].

Pancreatic ductal adenocarcinoma is a hypovascular tumor in a hypoxic microenvironment, and the major pathological feature is the high levels of fibrosis, termed desmoplasia, that generate an excessive interstitial fluid pressures at primary tumor sites and at metastatic ones [45]. Desmoplasia results in vasculature collapse that promotes cancer development and inhibits drug penetration and uptake inducing cancer resistance to targeting-therapy [46,47]. However, pancreatic ductal adenocarcinoma cells have high glucose uptake due to the presence of basal microvilli on the microvessels that represent a novel and unique pathological feature [48]. Despite the hypovascularization, pancreatic ductal adenocarcinomas are characterized by their strong capacity to proliferate and ability to metastasize.

By contrast, pancreatic neuroendocrine tumors are hypervascular tumors due to overexpression of angiogenic molecules such as VEGF and its related receptor (VEGFR), especially in liver metastasis [49,50,51]. In addition, it has been demonstrated that the serum levels of angiogenic cytokines, such as VEGF and IL-8, are associated with tumor progression in pancreatic neuroendocrine tumor patients, and they might use as biomarkers for prognosis and therapy [52].

For these reasons it is expected that anti-angiogenic therapy will be effective in both pancreatic ductal adenocarcinoma and pancreatic neuroendocrine tumors with the aim to block blood vessels growth to stop cancer cells growth (see Table 2 for an overview of anti-angiogenic drugs in pancreatic cancer) [19].

## 3. Anti-Angiogenic Inhibitors in Pre-Clinical Studies

At present, surgery is the gold standard treatment in localized pancreatic cancer, but when is not enough different drugs are used to decrease tumor proliferation and progression, and to treat tumor symptoms. In this section we discuss the pre-clinical studies of last 5 years concerning the most innovative anti-angiogenic approaches to pancreatic cancer (for the state of the art of previous years see [81,82]) focused on targeting therapy against specific angiogenic pathways, cellular and acellular components of tumor microenvironment, and specific cell surface markers.

Several Authors examined the effects of anti-angiogenic molecules on many model of human pancreatic ductal adenocarcinoma and pancreatic neuroendocrine tumors such as cells cultures, orthotopic or subcutaneous nude mouse and the newest genetically engineered mouse models for pancreatic ductal adenocarcinoma [83,84,85,86,87,88,89] and RIP-Tag2 mouse model for pancreatic neuroendocrine tumors [90,91].

Sunitinib is multitargeted tyrosine kinase inhibitor that targets both angiogenic pathways (VEGFR1-3, PDGFR) and pro-oncogenic pathways (c-kit, RET, stem-cell factor receptor and FMS-like tyrosine kinase-3) [92]. Sunitinib was explored in several pre-clinical and clinical studies for both pancreatic ductal adenocarcinoma and pancreatic neuroendocrine tumors. Wegner et al., in two pancreatic ductal adenocarcinoma xenograft models treated and untreated with sunitinib following changes by dynamic contrast-enhanced magnetic resonance imaging, provides sevidence that anti-angiogenic treatment with sunitinib induce changes in the tumor microenvironment, and furthermore, demonstrated that K^trans^ (vasculature functional parameter that combined effects of plasma blood flow, permeability and capillary surface area per unit mass) may be an adequate measure of tumor vascular density and hypoxia in pancreatic ductal adenocarcinoma [60,61]. For patients no-responding to gemcitabine-based treatments, sunitinib has been highlighted as a promising drug [62].

Other studies have been focused on molecules that explicate their anti-angiogenic activities against HIF-1α pathway, the major transcription factor expressed under hypoxia. Xu et al. hunting among 32 benzofuran derivatives that suppressed p53-independent tumor cells through inhibition of HIF-1 pathway, in an in vivo study on pancreatic ductal adenocarcinoma cell lines, showed that compound 90 suppressed tumor growth and exerted an anti-angiogenic activity [74].

As previously mentioned, pancreatic ductal adenocarcinoma growth, chemoresistance and metastasis are sustained by vasculogenic mimicry, an alternative type of blood supply independent of endothelial vessels and formed by highly invasive and genetically dysregulated cancer cells. Wei et al. demonstrated that verteporfin, a photosensitizer clinically used for photodynamic therapy to treat neovascularization, targeting the Hippo pathway, suppress the proliferation of human tumor cells, and tumor growth on the pancreatic ductal adenocarcinoma xenograft model [79]. Verteporfin arrests cells at the G1 phase, and induces apoptosis by activating the intrinsic apoptotic signalling pathway in dose- and time-dependent manner via reducing the expression of cyclin D1 and cyclinE1 [79]. Moreover, verteporfin inhibits tumor angiogenesis downregulating angiopoietin-2 (Ang-2) through inhibition of YAP activity, and suppress vasculogenic mimicry downregulating MMP2, VE-cadherin and α-SMA expression [79]. Tangutoori et al. enhanced the use of photosensitizing compounds combining them with biological therapeutic ones in the treatment of pancreatic ductal adenocarcinoma to induce tumor reduction [80]. In in vitro and in an in vivo subcutaneous mouse model of pancreatic ductal adenocarcinoma, they carried out a nanoscale intracellular drug delivery systems capable of multidrug delivery (verteporfin plus anti-VEGF monoclonal antibody bevacizumab) in a nano-photoactivatable liposome to enhance the efficacy photodynamic therapy combined with suppression of VEGF-mediated signalling pathways [80].

The transcription factor NF-κB is constitutively activated in pancreatic ductal adenocarcinoma and further activated by gemcitabine [93]. In athymic mice tumor growth model injected subcutaneously with IκBα-super-repressor or vector-expressing human pancreatic ductal adenocarcinoma cells, Waters et al. demonstrated that stable IκBα-super-repressor expression in vivo potentiated the antitumor effects of gemcitabine, resulting in decreased tumor growth in association with decreased cell proliferation [93].

VEGF, platelet derived growth factor (PDGF), FGF and their receptors are highly expressed in pancreatic ductal adenocarcinoma and their expression correlate with poor prognosis because they are involved in the development of resistance to anti-VEGF therapy and in the induction of metastasis [94,95]. Single-target anti-angiogenic agents have been studied for combined therapy in pancreatic ductal adenocarcinoma with limited success [96]. In 2015, Awasthi et al. investigated the antitumor activity of nintedanib, a triple angiokinase inhibitor that targets VEGFR1/2/3, FGFR1/2/3 and PDGFRα/β signalling, alone or in combination with Gemcitabine in two murine pancreatic ductal adenocarcinoma xenograft models [56]. They demonstrated a strong antitumor activity of nintedanib as a single agent and combined with the cytotoxic agent Gemcitabine [56]. In detail, nintedanib inhibits pancreatic ductal adenocarcinoma related cell proliferation and migration, blocks or downregulates signalling proteins such as PI3K/MAPK, induces apoptosis in pancreatic ductal adenocarcinoma associated stromal cells and in intratumoral ones, inhibits local tumor growth, enhances gemcitabine antitumor response, and reduces microvascular density [56].

The anti-angiogenic drug nintedanib has been proposed as a new treatment modality also for pancreatic neuroendocrine tumors patients. Bill et al. in nintedanib treated RIP-Tag2 mice, demonstrated a strong suppression of angiogenesis, accompanied by a reduced tumor burden, which translated into a significant prolongation of survival, without any impact on tumor lymphangiogenesis [57].

Pancreatic stellate cells are the principal cells responsible of desmoplasia in pancreatic ductal adenocarcinoma, display phagocytic activity and express toll-like receptors 2–5, which suggests a macrophage-like role in the pancreas for these cells [97,98,99]. Gonzalez-Villasana et al. demonstrated that nitrogen-containing bisphosphonates in combination with Nab-paclitaxel, which is known to enhance drug delivery in tumors, inhibited pancreatic stellate cells proliferation, their activation, the release of macrophage chemoattractant protein-1 and of type I collagen expression, and reduced angiogenesis [69].

A greater responses and improved antitumor results targeting tumor angiogenesis have been obtained using, nitrogen-containing bisphosphonates-paclitaxel in combination with Bevacizumab and Sunitinib [100].

Herrera et al. found the dual endothelin1/VEGF signal peptide receptor expressed by pancreatic ductal adenocarcinoma microvessels, cancer cells and cancer stem-like cells [70]. Moreover, dual endothelin1/VEGF signal peptide receptor-inhibition decreased angiogenesis, invasiveness, cancer stem-like cells-survival [70].

MicroRNAs regulate tumor cell proliferation, angiogenesis, and metastasis, and therefore they constitute highly promising targets for antitumor therapies [101]. Passadouro et al., using tumor cell line as an in vitro model of pancreatic ductal adenocarcinoma, demonstrated that the combination of microRNA silencing miR-21 with low amounts of sunitinib resulted in a strong and synergistic antitumor effect [61]. In fact, cell viability decrease of approximately 45%, which was much higher than that observed with any of the two strategies by themselves [61].

Sustained angiogenesis and immunosuppression are hallmarks of cancer and these processes share some regulators in physiological and cancer processes [102,103]. Ongoing clinical immunotherapeutic trials are based on the direct stimulation of the immune system [104,105]. Direct stimulation of the immune system with immune check-point inhibitors, such as antibody against programmed death protein 1/programmed death ligand, target also pro-angiogenic cytokines in addition to the tumor itself, making anti-angiogenic immunotherapy more resistant to immune-escape mechanisms.

In this context, Allen et al. demonstrated that treatment with anti-VEGFR2 and anti–programmed cell death ligand 1 antibodies induced high endothelial venules in RIP1-Tag2 transgenic mouse model of pancreatic neuroendocrine tumors that in turn promoted lymphocyte infiltration and activity through activation of lymphotoxin β receptor signalling [72]. Anti–programmed cell death ligand 1 therapy could make tumor susceptible to anti-angiogenic therapy and prolong its efficacy, and conversely, anti-angiogenic therapy could improve anti-programmed cell death ligand 1 treatment specifically when it generates intratumoral high endothelial venules that facilitate enhanced cytotoxic T cells infiltration, activity, and cancer cells destruction [72].

Several studies demonstrated the efficacy of anti-VEGF-A therapies in animal models of pancreatic cancer [81,82]. There are several modalities of disrupting the VEGF-A signalling including blocking VEGF-A secretion from tumors cells, neutralizing the VEGF-A ligand, blocking VEGF-A binding to VEGFRs, and blocking downstream signalling of the VEGFR. Keklikoglou et al., by genetic deletion of periostin (a matricellular protein expressed by stromal cells) in RIP1-Tag2 mice blunted tumor, demonstrated that revascularization and progression of pancreatic neuroendocrine tumors under extended VEGFA blockade are dependent on it [106]. Moreover, they showed that periostin deficiency also impeded the upregulation of FGF2, an adaptive mechanism previously implicated in pancreatic neuroendocrine tumors evasion from anti-angiogenic therapy [106].

The infiltration of tumor associated macrophages in pancreatic neuroendocrine tumors might correlate with tumor progression and metastasis formation. Krug et al. investigated the effect of targeted tumor associated macrophage therapy in vitro and in vivo using liposomal clodronate [67]. By immunohistochemistry and tissue-micro-array, they assessed that clodronate arrests tumor progression and reduce tumor angiogenesis in the RIP1-Tag2 mice [67]. However, combined therapy with liposomal Clodronate and the anti-angiogenic tyrosine kinase inhibitor sunitinib did not show any synergistic effects [67].

**Figure 1:**
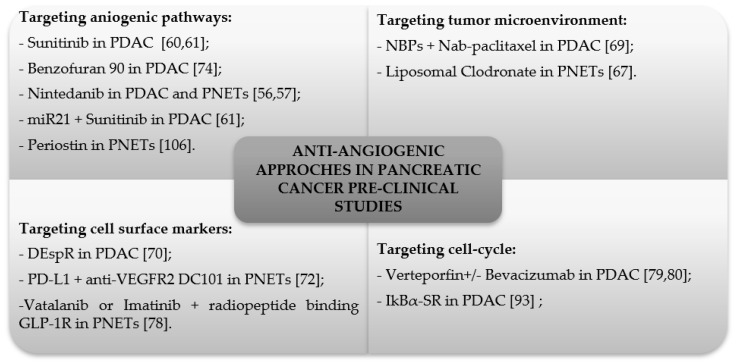
Anti-angiogenic approaches in pancreatic cancer pre-clinical studies of last 5 years discussed in the text.

Pancreatic neuroendocrine tumors are well vascularized and express somatostatin receptors and the glucagon-like peptide-1 receptor. Wicki et al., using the RIP1-Tag2 mice, demonstrated the potential benefit of co-administration of anti-angiogenic treatment with oral vatalanib (an inhibitor of VEGFR) or imatinib (a c-kit/PDGFR inhibitor) and radiotherapy with the a radiopeptide that selectively binds to glucagon-like peptide-1 receptor expressed on pancreatic neuroendocrine tumor cells [78]. Figure 1 summarizes the clinical studies of the past 5 years discussed in this section.

## 4. Anti-Angiogenic Inhibitors in Clinical Studies

The U.S. Food and Drug Administration and the European Medicines Agency have approved a number of angiogenesis inhibitors to treat cancer and several clinical studies, in search of biomarkers that allow to better characterize the different types of pancreatic cancer in order to improve patient’s management. In this section we discuss the most relevant clinical studies of last 5 years concerning the anti-angiogenic therapies for pancreatic cancers.

Hypoxia is one of the most potent inducers of an angiogenic response as a primary regulator of the angiogenic switch [107]. Tumor hypoxia activates anaerobic metabolism, angiogenesis, erythropoiesis and cell survival [108]. Lactate dehydrogenase (LDH), a key enzyme in the conversion of pyruvate to lactate during anaerobic metabolism, has been correlated with mechanisms underlying tumor hypoxia and angiogenesis [68,109,110]. Faloppi et al., in a randomised phase II trial for patients with locally advanced, unresectable or metastatic pancreatic cancer, treated with sorafenib, an anti-angiogenetic multitarget tyrosine kinase inhibitor, in combination with gemcitabine vs. gemcitabine alone demonstrated that LDH is a prognostic and predictive parameter to select candidate to receive sorafenib [58]. In patients with a lower LDH serum level under the inferior normal rate, this parameter was correlated to a better prognosis in terms of median progression-free survival (PFS) and overall survival (OS) [58]. Moreover, the patients with low LDH serum level treated with sorafenib plus gemcitabine showed an advantage in PFS and OS compared to patients treated with gemcitabine alone [58].

In pancreatic cancer, gemcitabine is one of the standard chemotherapy drugs used alone or in combination with other chemotherapy drugs to make treatment more effective. OS has been significantly prolonged with combined therapies but, considering the several side effects (skin rash, febrile neutropenia, and peripheral neuropathy/myelosuppression) of these therapies, new therapeutic protocols are always in progress. Yamaue et al., based on the promising results of their phase I trial, conducted a multicenter, randomized, placebo-controlled, double-blind phase II/III clinical trial to testing the combined therapy gemcitabine plus elpamotide to treat adenocarcinoma or adeno-squamous pancreatic cancer [73]. Elpamotide is an epitope peptide derived from the amino acid sequence of VEGFR2, previously, characterized as agent for anti-angiogenic immunotherapy against cancer in clinical settings [111]. Elpamotide induces cytotoxic T lymphocytes with potent cytotoxicity that are capable of killing VEGFR2-expressing human endothelial cells and in turn could reduce angiogenesis [111]. In the clinical trial, OS was 8.36 months for the active group and 8.54 months for the placebo group, but despite the lack of benefit in OS, subgroup analysis suggested that the patients who experienced severe injection site reaction, such as ulceration and erosion, might have better survival [73].

The efficacy of mammalian target of rapamycin (mTOR) inhibitors in combination with gemcitabine chemotherapy has been also evaluated [112]. Joka et al. pointed out an open-label, multicenter phase I study designed to determine the maximum tolerated dose and dose-limiting toxicity (DLT) of escalating doses of everolimus (a selective inhibitor of mTOR downstream signalling) plus gemcitabine in patients with advanced or metastatic pancreatic adenocarcinoma [75]. They found the maximum tolerated dose of a low-dose Gemcitabine treatment in combination with everolimus and no new safety concerns.

Another way to fight pancreatic cancer is combined chemo- and radiation therapy with targeted anti-angiogenic drugs, such as bevacizumab and sorafenib [54,55,113,114]. Chiorean et al. in a phase I study evaluated the safety and efficacy effects of sorafenib with gemcitabine-based chemoradiotherapy in locally advanced pancreatic adenocarcinoma, through pharmacodynamics analysis of tumor perfusion and vascularity and analysis of VEGFA and VEGFR2 single nucleotide gene polymorphisms [59].

Despite the promising results of the pre-clinical studies suggesting VEGF as a therapeutic target in pancreatic cancer, phase III trials of gemcitabine plus anti-angiogenic therapy with bevacizumab or axitinib (a VEGFR inhibitor) failed to reach their primary endpoint of OS [53,63,83,115]. Therefore, to date we are still looking for biomarkers that allow to identify subsets of patients who may benefit from these targeted anti-angiogenic therapies. Pant et al. evaluated the utility of bevacizumab-related hypertension as a biomarker for bevacizumab efficacy in pancreatic adenocarcinoma [116]. They evaluated the clinical outcomes according to bevacizumab-related hypertension using pooled data from 4 prospective studies of gemcitabine plus bevacizumab therapy. Their data demonstrated that pancreatic adenocarcinoma patients with any grade derive benefit from bevacizumab, allowing to consider bevacizumab-related hypertension as potential pharmacodynamic biomarker for the enrolment of patients to treat with this therapy. Patients had a significantly improved median OS (13.1 vs. 8.1 months), median time to tumor progression (7.6 vs. 5.5 months), objective response rate (ORR) (47% vs. 16%), and disease control rate (85% vs. 59%) [116].

Endostatin, a COOH-terminal fragment of collagen XVIII, is an inhibitor of endothelial proliferation and causes regression of large tumor to microscopic size inhibiting angiogenesis [117,118]. For these therapeutic potentials, it has been developed a recombinant human endostatin, namely endostar [119]. In non-small cell lung cancer patients, it was showed that endostar plus paclitaxel-carboplatin improved objective response rate (ORR) and exhibited a good safety profile [120]. Cheng et al. in a single-center phase II trial, assessed the effect of endostar and temozolomide or dacarbazine plus 5-fluoruracile in 14 patients with locally advanced or metastatic well-differentiated pancreatic neuroendocrine tumors checking the radiographic response rate [71]. They observed an objective radiographic response rate of 46% among patients treated with endostar combined with chemotherapy, suggesting that the study regimen could effectively reduce the tumor burden (ORR was 33% and PFS was 12 months).

The multitargeted kinase inhibitor sunitinib has been approved for the treatment of well-differentiated pancreatic neuroendocrine tumors because it showed a prolonged PFS and a trend in benefit in OS [121]. Pellat et al. in a prospective phase II trial evaluated the potential predictive biomarkers of sunitinib activity in patients affected by grade 3 gastro-entero-pancreatic neuroendocrine tumors [64]. Following PDGFRβ, carbonic anhydrase 9, Ki-67, VEGFR2 and p-AKT as potential biomarkers by immunohistochemistry, they found that sunitinib displayed a greater activity in grade 3 gastro-entero-pancreatic neuroendocrine tumors with low Ki-67 and a lower one when p-AKT expression was high [64].

Among multitargeted kinase inhibitors, pazopanib was approved in monotherapy for a phase II study in metastatic gastro-entero-pancreatic neuroendocrine tumors showing an ORR of 18.9%, a disease control rate of 75.7% and a median PFS of 9.1 month [122]. Grande et al. in a multicenter, open-label, phase II study (PAZONET study) assessed the activity of Pazopanib in previously treated advanced neuroendocrine tumors, including patients who received mTOR inhibitors and other multitargeted agents [65]. They showed a Pazopanib clinical activity in patients with advanced PNETs regardless of previous treatments. In fact, two thirds of the patients had a clinical benefit rate at 6 months with a median PFS of 9.5 months [65]. Additionally, they correlated the activity of pazopanib to circulating tumor cells and soluble-s VEFGR2-3 gene polymorphisms that may constitute potential biomarkers to predict response of individual patients to pazopanib [65].

Bevacizumab, used as a tumor-starving therapy, has also been studied in patients with pancreatic neuroendocrine tumors. Yao et al. in a randomized phase III study of octreotide (a synthetic somatostatin analogues) in combination with bevacizumab or pegylated interferon alpha-2b (IFNα-2b) showed an ORR of 12% with octreotide/bevacizumab compared to 4% with octreotide/IFNα-2b, and a median PFS of 16.6 months in the first group and 15.4 months in the second one [77]. Although no improvement in PFS was observed, bevacizumab was associated with a higher ORR, a longer time to treatment failure, and a lower rate of fatigue than IFN [77].

Bevacizumab efficacy and safety was also evaluated in combination with pertuzumab, a humanized monoclonal antibody that binds to the extracellular domain II of the HER2- receptor, blocking its ability to dimerize with other HER receptors (EGFR (HER1), HER3 and HER4). Therefore, pertuzumab inhibits the MAPK and PI3K pathways with a mechanism of action distinct from other tyrosine kinase inhibitors [123]. Bendall et al. in a phase II study of the combination of bevacizumab, pertuzumab, and octreotide long-acting release for pancreatic neuroendocrine tumors patients, showed some signs of clinical efficacy and good tolerability [66]. The median PFS was 6.5 months and OS was 26.4 months with a 16% ORR [66].

Somatostatin analogues have emerged as a successful tool for the management of neuroendocrine diseases. Somatostatin analogues inhibit hormonal secretion by binding to their receptor and thus provide relief of symptoms in patients with functional pancreatic neuroendocrine tumors [125,126,127]. Sampedro-Núñez in human primary and metastatic gastro-entero-pancreatic neuroendocrine tumors studied the presence and potential functional roles of truncated variants of somatostatin receptors, and their relationships with the angiogenic system Ang/Tie-2 and VEGF by Real Time-PCR, immunofluorescence and immunohistochemistry [124]. Indeed, the functional role of truncated variants of somatostatin receptor was analyzed in gastro-entero-pancreatic neuroendocrine tumors cell lines [124]. They demonstrated that truncated variants of somatostatin receptor are overexpressed in gastro-entero-pancreatic neuroendocrine tumors and is associated to enhanced aggressiveness, suggesting its potential value as biomarker and target in gastro-entero-pancreatic neuroendocrine tumors [124]. Clinical studies in the past 5 years discussed in this section are summarized in Table 3.

## 5. Concluding Remarks

It is clear from the recent literature that, despite the increasing number of new anti-angiogenic therapies, there are few data regarding new targeted agents compared to others and studies focusing on the optimal sequencing strategy or a combined approach to have the best long-term outcome. Instead, the look for biomarkers for personalized therapy and new therapeutic approaches against the cellular and acellular components of the microenvironment is evident. The proper patient selection through predictive and prognostic biomarkers may maximize the efficacy of anti-angiogenic therapy in cancer [59].

The aim of anti-angiogenic therapy is to reduce tumor blood vessel increase in order to inhibit tumor growth, reducing oxygen and nutrients supplying. However, long term anti-angiogenic therapy sometimes leads to tumor hypoxia that in turn triggers VEGF production, genetic instability in tumor endothelial cells and vascular permeability [128,129].

Moreover, the anti-angiogenic therapy negatively affects the drug delivery. Drugs transport to cancer cells is inefficient due to structurally and functionally abnormal blood vessels morphology [130]. Tumor blood vessels are fenestrated with a thin wall due to the lack of pericytes changing the permeability within the same tumor and between different tumors [130,131]. Anti-angiogenic therapies are ineffective because tumor vascularisation may occur via alternative mechanisms, which include vessel co-option, vasculogenic mimicry and vasculogenesis [21]. This partially explain the necessity of combined inhibition of angiogenesis and alternative mechanisms in treatment of pancreatic cancer [79,80].

Probably, tumor blood vessels should not inhibit tumor starvation and shrinkage, but they should normalize to enhance effective drug concentration. Nagathihalli et al. targeting inhibition of STAT3 combined with gemcitabine in tumor cell lines, enhanced drug delivery, therapeutic response, and favoring blood vessel normalization [132,133].

The new therapies should have to experiment the timing of chemotherapy. Chemotherapy before surgery may help to shrink the tumor and to destroy undetectable micrometastases reducing the risk of relapse after surgery. Another possibility may be chemotherapy before and after surgery.

Further studies are needed to have an accurate identification and characterization of the key regulators of angiogenesis to develop new therapies to simultaneously target different molecular signalling pathways. Moreover, it will be necessary to focus on choosing the right drug based on the stage of the tumor, on the most effective combination therapies and on the study of the mechanisms of resistance. The future will be focused on the realization of a personalized anti-angiogenic therapy.

## Figures and Tables

**Table 1:** Pancreatic cancer risk factors.

Tobacco, Cigar and Pipe Use	20–30% of pancreatic cancers are caused by smoking.
Overweight and obesity	
Diet	Higher in red and processed meats and lower in fruits and vegetables.
Lack of physical activity	
Workplace exposure to chemicals	Chemicals used in the dry cleaning and metal working industries.
Age	Risk goes up as people age.
Gender	Men.
Race	African Americans more likely to develop than white.
Blood group	People with blood groups A, AB and B have a higher risk.
Family history	
Inherited genetic syndromes	Hereditary pancreatitis, Peutz-Jeghers syndrome, Familial malignant melanoma and pancreatic cancer, Hereditary breast and ovarian cancer syndrome, Lynch syndrome, Li-Fraumeni syndrome, Familial adenomatous polyposis.
Type 2 Diabetes	
Chronic pancreatitis	
Cirrhosis of the liver	
Gastric ulcer	*Helicobacter pylori* related.

Data from American Cancer Society (Last Revised: May 2016), Pancreatic Cancer UK (Last Revised: January 2018), and Cancer. Net Editorial Board (Last Revised: May 2018).

**Table 2:** Drugs targeting directly or indirectly angiogenesis in pancreatic cancer.

Drugs Targeting Growth Factor- and Growth Factor Receptor Inhibitors	Drug Target/Mechanism of Action	Pre-Clinical/Clinical Studies	Evaluated in PDAC/PNETs
Axitinib [53]	Selective inhibitor of VEGFR 1–3	Clinical	PDAC
Bevacizumab [54,55]	Humanized anti-VEGF monoclonal antibody	Clinical	PDAC
Nintedanib [56,57]	Triple angiokinase inhibitor that targets VEGFR 1–3. FGFR 1–3 and PDGFRα/β	Pre-clinical	PDAC and PNETs
Sorafenib [58,59]	Small molecule inhibitor of Raf kinase, PDGF, VEGFR 2–3 and c-kit	Clinical	PDAC
Sunitinib [60,61,62,63,64]	Multitargeted tyrosine kinase inhibitor that targets VEGFR1-3, PDGFR, c-kit, RET, stem-cell factor receptor and FMS-like tyrosine kinase-3	Pre-clinical and Clinical	PDAC and PNETs
Pazopanib [65]	Multitargeted tyrosine kinase inhibitor that targets VEGFR, PDGFR and c-kit	Clinical	PNETs
Pertuzumab [66]	Humanized anti-HER2-receptor	Clinical	PNETs
**Drugs targeting the tumor stroma**			
Liposomal Clodronate [67,68]	Arrest tumor progression and angiogenesis	Pre-clinical	PNETs
Nitrogen-containing bisphosphonates (NBPs) [69]	Inhibit PSCs proliferation and activation, the release of MCP-1, type I collagen expression	Pre-clinical	PDAC
**Drugs targeting cancer stem cells**			
DEspR [70]	DEspR-inhibition decrease angiogenesis, invasiveness, CSCs-survival and anoikis resistance	Pre-clinical	PDAC
Endostar [71]	Inhibit endothelial proliferation	Clinical	PNETs
**Drugs targeting immune-cells**			
Anti-PD-L1 [72]	Promote lymphocyte infiltration and activity through activation of LTβR signalling	Pre-clinical	PNETs
Elpamotide [73]	Induce CTLs to kill VEGFR2-expressing endothelial cells	Clinical	PDAC
**Drugs targeting other molecules or pathways**			
Benzofuran 90 [74]	Suppress p53-indipendent tumor cells through inhibition of HIF-1 pathway. Inhibits tumor angiogenesis downregulating Ang-2 through inhibition of YAP activity, and suppress VM downregulating MMP2, VE-cadherin and α-SMA expression	Pre-clinical	PDAC
Everolimus [75]	Selective inhibitor of mTOR downstream signalling	Clinical	PDAC
IkBα-SR [76]	Inhibit NF-κB pathway	Pre-clinical	PDAC
miR21 [61]	Decrease cell viability	Pre-clinical	PDAC
Octreotide [77]	Synthetic somatostatin analogues that inhibit hormonal secretion	Clinical	PNETs
Radiopeptide anti-GLP-1R [78]	Target beta-cells with radiopepetide	Pre-clinical	PNETs
Verteporfin [79,80]	Arrest cell-cycle and induce apoptosis targeting Hippo pathway	Pre-clinical	PDAC

**Table 3:** Main clinical studies ongoing in pancreatic cancer of last 5 years discussed in the text.

Drug’s Name	Sample Size	Combined Regimens	Response Rate	Objective Response Rate	Overall Survival (Months)	Progression Free Survival (Months)	Median Survival Time	Didease Control Rate	Time to Progression
Sorafenib [58]	71	Gemcitabine plus or not Sorafenib in patients with LDH values under or above the cut-off			10.7 vs. 5.9	5.2 vs. 2.7			
Elpamotide [73]	153	Gemcitabine plus or not Elpamotide			No significative	3.71 vs. 3.75	8.36 vs. 8.54	59.6% vs. 60.4%	
Everolimus [75]	27	Everolimus in combination with escalating low-dose gemcitabine	13%						
Sorafenib [59]	25	Gemcitabine-based chemoradion therapy plus or not Sorafenib			12.6 in evaluable patients; 11.5 in intent to treat patients; 21.6 in patients with VEGF-A -2578 AA, -1498 CC genotypes; 14.7 in patients with VEGF-A -1154 AA genotype	10.6 in evaluable patients; 9.9 in intent to treat patients			10.7 in evaluable and in intent to treat patients
Bevacizumab [116]	163	Gemcitabine plus Bevacizumab in patient with related hypertension (B-HTN) or not		47% vs. 16%	13.1 vs. 8.1			85% vs. 59%	
Endostar [71]	14	Endostar and Temozolomide or Dacarbazine plus 5-fluorouracil	46%	33% vs. 50% in no therapy vs. prior therapy	Not reached	12			
Sunitinib [64]	26	Sunitinib after different chemotherapy protocols	58%		6.0	1.4			
Pazopanib [65]	44	Pazopanib			24.1	9.5			
Octreotide [77]	427	Octreotide vs. Octreotide plus Bevacizumab or interferon alfa-2b		12% in Bevacizumab arm; 4% in interferon alfa-2b arm	No significative	16.6 in Bevacizumab arm; 15.4 in interferon alfa-2b arm			
Pertuzumab [66]	43	Bevacizumab, Pertuzumab and Octreotide		16%	26.4	6.5			
sst5TMD4 [124]	42	Study designed to focus on functional roles of somatostatin receptors							
